# Supplementary material for: Evaluation of Antibody Kinetics Following COVID-19 Vaccination in Greek SARS-CoV-2 Infected and Naïve Healthcare Workers
Source: J Pers Med. 2023 May 29;13(6):910. doi: 10.3390/jpm13060910 (PMC10301969; doi:10.3390/jpm13060910)
Supplement: Supplementary file 1 [file jpm-13-00910-s001.zip › Table S1.pdf]

Table S1. Association of the time from the second vaccine dose to first SARS-CoV-2 infection and of the time from the second vaccine dose to SARS-CoV-2 reinfection with co-morbidities.

	Time from the second vaccine dose to first SARS-CoV-2 infection			
	Univariate analysis		Multivariate analysis	
	Unstandardized b (95% CI)	P-value	Unstandardized b (95% CI)	P-value
Age	0.033 (-0.005 – 0.070)	0.088	0.035 (-0.007 – 0.078)	0.105
Sex	-0.167 (-1.150 – 0.815)	0.737	-0.019 (-1.020 – 0.982)	0.971
Obesity	-0.717 (-1.962 – 0.528)	0.258	-1.075 (-2.372 – 0.222)	0.104
Hypertension	0.634 (-0.563 – 1.831)	0.298	0.008 (-1.513 – 1.530)	0.991
Dyslipidemia	0.732 (-0.439 – 1.903)	0.220	0.290 (-1.180 – 1.760)	0.698
Diabetes mellitus	0.436 (-1.419 – 2.292)	0.644	-0.047 (-2.103 – 2.008)	0.964
CAD	2.904 (-0.705 – 6.513)	0.114	2.451 (-1.337 – 6.238)	0.204
	Time from the second vaccine dose to SARS-CoV-2 reinfection			
	Univariate analysis		Multivariate analysis	
	Unstandardized b (95% CI)	P-value	Unstandardized b (95% CI)	P-value
Age	0.128 (0.005 – 0.250)	**0.041**	0.192 (0.044 – 0.341)	**0.013**
Sex	2.245 (-0.891 – 5.381)	0.154	1.985 (-1.342 – 5.313)	0.230
Obesity	-0.634 (-4.122 – 2.853)	0.713	-3.975 (-8.362 – 0.413)	0.074
Hypertension	0.713 (-3.607 – 5.034)	0.738	3.474 (-6.868 – 13.816)	0.495
Dyslipidemia	0.906 (-2.881 – 4.692)	0.629	-0.982 (-9.337 – 7.373)	0.810
Diabetes mellitus	1.984 (-7.018 – 10.986)	0.656	5.936 (-6.556 – 18.429)	0.336
CAD	-2.110 (-8.555 – 4.335)	0.509	-4.675 (-12.788 – 3.438)	0.246

CI, confidence interval; CAD, coronary artery disease. Bold values indicate statistically significant values ($P < 0.05$).
