# Supplementary material for: Evaluation of Antibody Kinetics Following COVID-19 Vaccination in Greek SARS-CoV-2 Infected and Naïve Healthcare Workers
Source: J Pers Med. 2023 May 29;13(6):910. doi: 10.3390/jpm13060910 (PMC10301969; doi:10.3390/jpm13060910)
Supplement: Supplementary file 1 [file jpm-13-00910-s001.zip › Table S2.pdf]

Table S2. Univariate and multivariate logistic regression analysis demonstrating the associations between SARS-CoV-2 reinfection and co-morbidities.

	SARS-CoV-2 reinfection			
	Univariate analysis		Multivariate analysis	
	Odds ratio (95% CI)	p-value	Odds ratio (95% CI)	p-value
Age	0.975 (0.951 – 0.997)	**0.046**	0.956 (0.927 – 0.986)	**0.004**
Sex (male)	1.158 (0.592 – 2.267)	0.668	0.991 (0.483 – 2.037)	0.981
Obesity	0.508 (0.234 – 1.101)	0.086	0.434 (0.179 – 1.050)	0.064
Hypertension	0.728 (0.313 – 1.693)	0.461	0.871 (0.260 – 2.915)	0.823
Dyslipidemia	0.606 (0.288 – 1.273)	0.186	0.575 (0.202 – 1.631)	0.298
Diabetes mellitus	0.995 (0.231 – 4.280)	0.995	1.608 (0.312 – 8.277)	0.570
CAD	0.342 (0.039 – 2.472)	0.232	0.183 (0.024 – 1.637)	0.126

CI, confidence interval; CAD, coronary artery disease. Bold values indicate statistically significant values ($P < 0.05$).
